# ID 40 - Diagnóstico Situacional de Atividades em Avaliação de Tecnologias em Saúde nas Secretarias Estaduais de Saúde

**DOI:** 10.5327/2237-9622.2025.v34s1.40

**Published:** 2025-11-25

**Authors:** Ana Paula Blankenheim, Bruna Marmett, Rodrigo Pereira de Almeida, Bárbara Cristiane da Silva, Roseana Boek Carvalho, Marina Petrasi Guahnon, Muriel Primon de Barros, Gilson Pires Dorneles, Suena Medeiros Parahiba, Maicon Falavigna

**Keywords:** diagnóstico situacional, gestão em saúde, educação em saúde

## Abstract

**Introdução:**

No contexto estadual do Sistema Único de Saúde (SUS), a Avaliação de Tecnologias em Saúde (ATS) possui particularidades que envolvem a implementação de tecnologias em saúde nos territórios, além de integrar as incorporações em saúde publicadas pelo Ministério da Saúde (MS) com os fluxos de atenção já existentes. Devido às peculiaridades e às necessidades locais, as Secretarias Estaduais de Saúde podem desenvolver seus próprios protocolos clínicos e outros produtos em ATS. Todavia, ainda não foi totalmente explorada essa realidade. Dessa forma, o objetivo deste trabalho foi realizar um diagnóstico situacional sobre as atividades em ATS nas SES.

**Método:**

O diagnóstico situacional foi realizado a partir de um questionário elaborado em formato de planilha, contemplando 34 perguntas, distribuídas em sete domínios: Identificação, Regulamentação, Atividades de ATS na SES, Suporte ao Judiciário, Atividades de ATS afins à Conitec, Acesso à informação e estrutura física e Dificuldades na aplicação. As informações de cada SES seriam preenchidas em relação ao período de 2023 a 2024 por até dois servidores da SES. O questionário e suas orientações foram enviadas por e-mail. Os dados obtidos foram apresentados de forma descritiva.

**Resultados:**

Das 27 SES contatadas, 26 participaram do diagnóstico situacional. Entre elas, oito (30,8%) relataram ter um Nats em sua constituição formal (Bahia, Ceará, Goiás, Mato Grosso, Rio Grande do Norte, Rondônia, São Paulo, Espírito Santo), quatro (15,4%) informaram possuir um centro de atividades em ATS com denominação ou formalização diferente (Alagoas, Distrito Federal, Goiás, Santa Catarina), 11 (42,3%) SES apresentavam uma CFT (Ceará, Distrito Federal, Espírito Santo, Maranhão, Mato Grosso, Minas Gerais, Pernambuco, Piauí, Rio Grande do Norte, Roraima, Tocantins) e nove (34,6%) SES relataram não possuir um grupo de ATS formalizado ou ativo (Acre, Amapá, Amazonas, Mato Grosso do Sul, Pará, Paraíba, Paraná, Rio de Janeiro, Sergipe). Treze (50,0%) SES relataram ter desenvolvido algum produto de ATS nos anos de 2023 e 2024. Nenhuma SES declarou ter solicitado incorporação de tecnologias diretamente para a Conitec no período, enquanto oito (30,8%) SES declararam que realizaram contribuição em consultas públicas da Conitec.

**Conclusão:**

O diagnóstico situacional das atividades em ATS em SES é um passo importante para a compreensão das realidades locais e planejamento de ações coordenadas de promoção do tema. Visto que as SES com formalização de Nats são as que mais desenvolvem produtos em ATS, esse marco regulatório apresenta-se como crucial para o desenvolvimento da ATS em âmbito estadual. A ampliação e a capacitação técnica dos recursos humanos, assim como a disponibilização de recursos financeiros e de infraestrutura, podem gerar avanços na qualificação de produtos em ATS em diversas SES e fortalecer o engajamento em ações federais.

